# Pilot Study of Blood Perfusion Changes at PC4 and Its Surrounding Points Induced by Acupuncture and Moxibustion

**DOI:** 10.1155/2021/2431570

**Published:** 2021-11-26

**Authors:** Shuyong Jia, Qizhen Wang, Hongyan Li, Xiaojing Song, Shuyong Wang, Weibo Zhang, Guangjun Wang

**Affiliations:** ^1^Institute of Acupuncture and Moxibustion, China Academy of Chinese Medical Sciences, Beijing, China; ^2^Institute of Basic Research in Clinical Medicine, China Academy of Chinese Medical Sciences, Beijing, China

## Abstract

Acupuncture and moxibustion are widely used in clinical practice; however, the differences between their mechanisms are unclear. In the present study, the response of blood perfusion resulting from acupuncture or moxibustion at Ximen (PC4) and its surrounding points was explored. Using the wavelet method, the differences in the frequency interval of blood flux were observed. Furthermore, the correlations between these points were analyzed. The results suggested that moxibustion could significantly improve blood flow perfusion at PC4 compared to acupuncture; however, there was no significant difference around PC4. The response of blood flux at PC4 to different stimulations was related to the frequency V (0.4–1.6 Hz) component. However, a difference in response at other points was not observed. Correlation analysis showed that both acupuncture and moxibustion could cause a decline in the correlation of blood flux signals at these recorded points, but there was no significant difference between these techniques. The results suggested that, at least in the forearm, the acupuncture or moxibustion only influenced the level of blood perfusion locally.

## 1. Background

Acupuncture has been widely used in clinical practice since 2500 years. Traditionally, acupuncture is performed with needles that are manipulated by the hands, while moxibustion produces thermal stimulation effects by burning moxa. Although acupuncture and moxibustion are different in clinical practice, they are believed to have similar clinical outcomes. However, a recent study has indicated that moxibustion and electroacupuncture have different effects on special-type irritable bowel syndrome [1, 2]. Further studies have shown that electroacupuncture and moxibustion have different effects on the network within the brain [3]. Significant basic research also supports that electroacupuncture and moxibustion have different effects [4]. Since the effects of acupuncture and moxibustion are different, different responses can be obtained at the regional acupoint.

Our previous study has shown that the local blood flux at the Weishu acupoint (BL21) is significantly increased after moxibustion compared to that after acupuncture, and there are many frequency intervals involved in the regulation of blood perfusion [5]. This shows that the regulation effect of moxibustion on the blood flow at BL21 is multifactorial. However, it is unclear whether acupuncture or moxibustion can regulate blood flow at acupoints in other parts of the body in the same way. In addition, it is unclear whether blood flow regulation in the area adjacent to the stimulation site is consistent with that at the stimulation site. Therefore, this study aimed to analyze the difference between acupuncture and moxibustion by detecting changes in blood flow at PC4 and the surrounding regions.

## 2. Methods

### 2.1. Participants

Ten healthy participants (age: 28 ± 4 years; four men and six women) were recruited. None of the participants had any diseases or were taking any medication that would affect their cardiovascular function. All participants were requested to avoid consuming alcohol, tea, or coffee at least 24 h prior to the test. Each participant agreed to undergo two interventions—acupuncture or moxibustion. The order of interventions was randomly determined. The interval between the two interventions was >1 week.

### 2.2. Ethics Approval and Consent

This study was approved by the Institutional Research Ethics Board of Acupuncture and Moxibustion, China Academy of Chinese Medical Sciences. In accordance with the Declaration of Helsinki, each participant provided informed consent and had an adequate understanding of the procedure and purpose of this study.

### 2.3. Protocol for Measurement of Blood Perfusion

**Figure 1:**
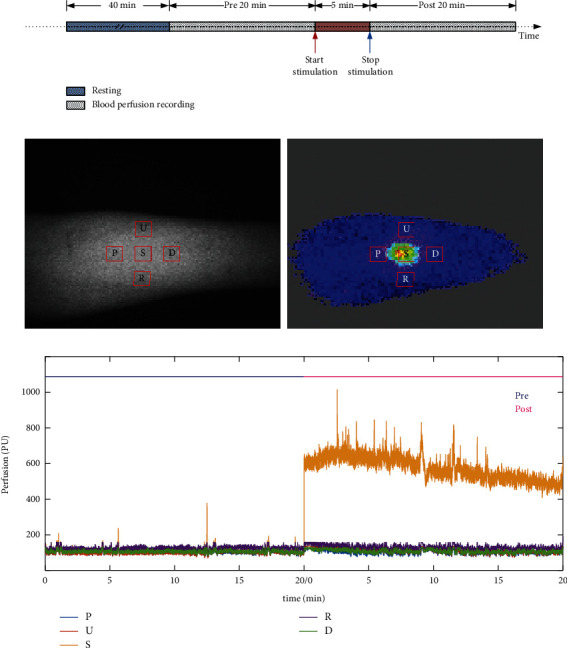
Study design and blood flux recording points. (a) Stimulation flow diagram: participants rest for 40 min before recording the baseline value for 20 min. After acupuncture or moxibustion for 5 min, blood flux is recorded for 20 min. (b) Recording points of the right forearm: left image is a photograph of the recording site and right image is a blood flow diagram. S is PC4, which receives stimulation. Both point P (proximal side) and point D (distal side) are on the pericardium meridian. Point R is on the radial side of the pericardium meridian, while point U is on the ulnar side. Point S and the other four points are equidistant. (c) Raw blood flux signals of recording points. Pre, prestimulation; post, poststimulation.

The measurement process can be found in Figure 1(a). The Ximen acupoint (PC4) was marked by a senior acupuncture doctor. PC4 is located at the “anterior aspect of the forearm region, between the tendons of the palmaris longus and the flexor carpi radialis, 5 B-cun proximal to the palmar wrist crease” [6]. In total, blood flux at five points was recorded (Figure 1(b)). Point S is PC4, points P (proximal side) and *D* (distal side) are on the pericardium meridian, point *R* is on the radial side of the pericardium meridian, point U is on the ulnar side, and point S and the other four points are equidistant. When determining the recording signal, the area of the region of interest (ROI) of the S point was determined first, and the area of the ROIs of the other four points was copied from that of the S point to ensure that the area of the ROIs of these five points was equal size.

In a previous study, the FLPI system (Moor Instruments, Devon, United Kingdom) was used to measure blood flux at these five points. The unit of blood flux is PU [7]. Before and after acupuncture-related stimulation, 20-min recordings were acquired at a 25 Hz sample rate (Figure 1(a)), with an exposure time of 8.3 ms [5].

All measurements and stimulations were performed in a temperature-controlled (24°C–26°C) laboratory. All participants were asked to maintain a comfortable position, and the skin of the forearm was sterilized. After a period of cardiovascular stability (40 min), baseline blood flux was recorded for 20 min. Subsequently, participants were stimulated by manual acupuncture or moxibustion at PC4, and the skin blood flux at this point was recorded for 20 min.

### 2.4. Protocol for Stimulation

Except the difference in acupoints in this study, the stimulation method was identical to that used in our previous study [5]. For acupuncture, after baseline recording, a small acupuncture needle (0.25 × 25 mm, Suzhou Dongbang Acup Inc., Suzhou, China) was inserted into the PC4 acupoint at a depth of 15 mm. To maintain the soreness and numbness sensation of De-Qi, the needle was slowly rotated for 5 min before stopping the intervention. For moxibustion, the ignited moxa roll was held approximately 2–3 cm above the PC4, which produced a mild warm and comfortable sensation for 5 min.

### 2.5. Blood Flux Analysis

Both time and frequency domain analyses were performed using MATLAB software (MathWorks Inc., Natick, MA, USA). The raw recording data file was opened with moor full-field laser perfusion imager (FLPI) review (V4.0, Moor Instruments, UK), and the related raw data were exported for further analysis in .txt format. For each recording point, the mean blood flux for 20 min was calculated before and after stimulation (Figure 1(c)).

The blood flow signal is a complex signal composed of multiple frequency components. Different frequency bands may reflect different physiological rhythms [8]. Thus, the blood flux signal can be analyzed in the frequency domain [9–12]. In the present study, wavelet analysis was performed on the blood flux signal (20 min) using a Morlet mother wavelet, as in our previous study [5].

### 2.6. Correlation Analysis of Blood Flux Signals between Different Points

To analyze the relationship between blood perfusion of the five recording points, moving Pearson correlation analysis of two time series was performed. The specific calculation method is based on the toolbox provided by Mack [13]. To evaluate the reliability of this method, MATLAB software was used to generate 10 pairs of random signals. Each signal contains 60000 data points, which is the same as the blood flux data of each recording point (Figure S). In the analysis of real data of blood flux, the size of the sliding window was set to 500 data points, and the moving Pearson correlation coefficient was calculated.

### 2.7. Statistical Analysis

Data are expressed as mean ± standard error. The level of significance was set at *p* < 0.05. Statistical analysis was performed using the paired *t* test with *R* software [14]. All reported *P* values were two sided.

## 3. Results

### 3.1. Mean Blood Flux at Different Points

**Figure 2:**
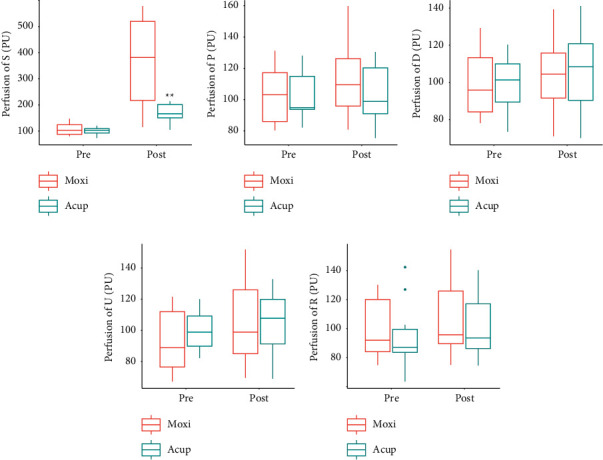
Mean of blood flux signals at different points. (a) Changes in skin blood flux at PC4 (^∗∗^, *p* < 0.01; Acup vs. Moxi, paired *t* test). (b) Changes in skin blood flux at the proximal point. (c) Changes in skin blood flux at the distal side point. (d) Changes in skin blood flux at the ulnar point. (e) Changes in skin blood flux at the radial point. S, PC4 point stimulated by acupuncture or moxibustion; P, proximal side points; D distal side point; R, radial side point; U, ulnar side point; Acup, acupuncture; and Moxi, moxibustion.

The changes in blood flux before and after stimulation are shown in Figure 2. Before stimulation, there was no significant difference in the mean blood flux at PC4 and its surrounding recording points (Figure 2(a)). After stimulation, the blood flux at PC4 induced by moxibustion was significantly higher than that induced by acupuncture. However, there was no significant change in the surrounding recording points (Figure 2(b)–2(e)).

### 3.2. Wavelet Transformation of Blood Flux at Different Points

**Figure 3:**
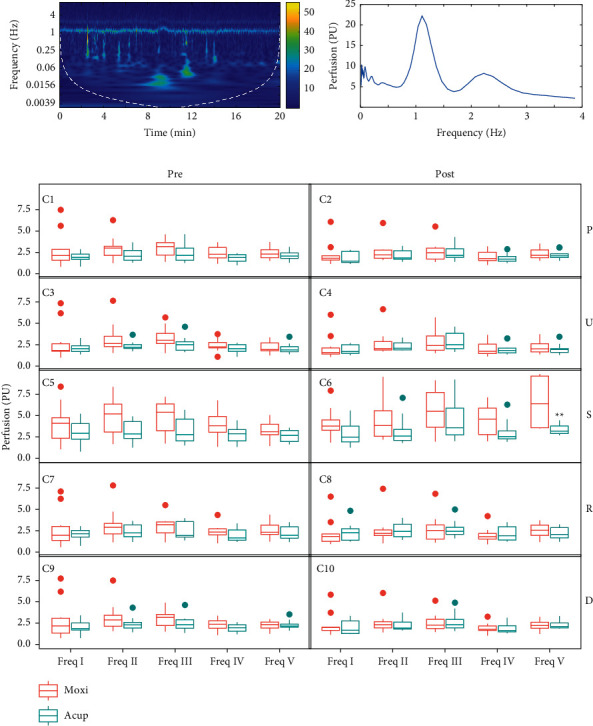
Morlet wavelet transformation results. (a) Time frequency results of a single participant. (b) Frequency-domain results. (c) Comparison of acupuncture and moxibustion stimulation. The left side shows the result before stimulation, and the right side shows the result after stimulation (^∗∗^, *p* < 0.01; Acup. vs. Moxi, paired *t* test). All values are reported as mean ± standard error. Freq, frequency; Freq I, 0.0095–0.02 Hz, Freq II, 0.02–0.06 Hz, Freq III, 0.06–0.15 Hz, Freq IV, 0.15–0.4 Hz, and Freq V, 0.4–1.6 Hz; Acup, acupuncture; and Moxi, moxibustion.

Blood flux signals were transformed using the Morlet mother wavelet (Figure 3(a)), and the frequency-domain results were further analyzed (Figure 3(b)). No significant difference was observed between acupuncture and moxibustion at different frequency intervals before stimulation (Figure 3(c)). After stimulation, the difference only appeared at PC4 (S), and the difference only appeared at frequency V (Figure 3(c)6). There was no significant difference in the other frequency intervals.

### 3.3. Correlation Analysis between Different Points

**Figure 4:**
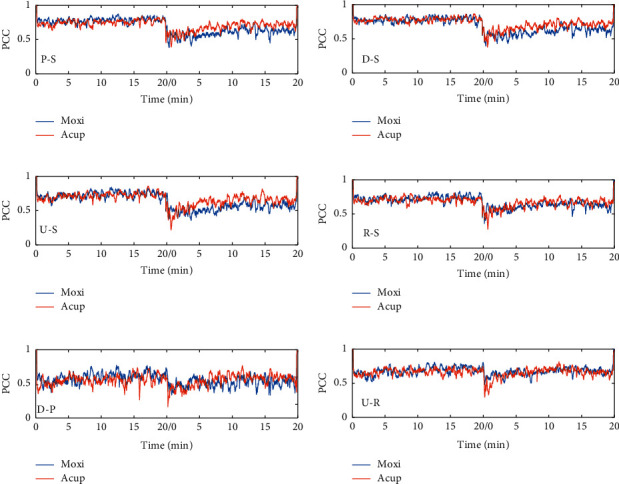
Blood flux signal correlation analysis between two points. (a) Correlation between the P point and S point. (b) Correlation between the D point and S point. (c) Correlation between the U point and S point. (d) Correlation between the R point and S point. (e) Correlation between the D point and P point. (f) Correlation between the U point and R point. PCC, Pearson correlation coefficient. The PCC is the average of 10 participants. Acup, acupuncture; Moxi, moxibustion.

In our data simulation, Pearson correlation coefficients were generated from 10 pairs of data. After averaging, the Pearson coefficient of the random signal fluctuated around 0. For forearm blood flux signals, the correlation coefficients between the surrounding points and PC4 were >0.5 at rest (Figure 4(a) and 4(b)), while the correlation coefficients of both D-P (Figure 4(e)) and U-R (Figure 4(f)) fluctuated around 0.5. Irrespective of acupuncture or moxibustion stimulation, the correlation coefficient decreased. However, there was no significant difference between acupuncture and moxibustion stimulation.

## 4. Discussion

In our previous study, changes in blood flow regulation at PC4 after different temperature stimulations were monitored in detail [15, 16]. However, there is a lack of data on blood flow changes caused by acupuncture or moxibustion. The current pilot study showed that there was a difference in the response of blood perfusion at PC4 after acupuncture or moxibustion stimulation. To our knowledge, this is the first study to compare the vascular response at PC4 and its surrounding points after stimulation by correlation and wavelet analysis. Although previous studies have showed the local vascular response to acupuncture [17, 18], to our knowledge, this is the first study to detect the correlation between blood flux signals and PC4. Based on our study, acupuncture and moxibustion have different effects on the average blood flux at PC4, which is related to the frequency V interval (0.4–1.6 Hz), but no different effects on the surrounding points.

Blood flux oscillations from 0.0095 to 1.6 Hz may be related to different physiological rhythms [8], which can be divided into five intervals in the frequency domain [9–12]. Generally, wavelet analysis is used to separate frequency intervals, which provides a noninvasive method to explore the mechanisms of blood perfusion regulation. The current results showed the frequency V interval was different in local blood regulation after acupuncture or moxibustion stimulation, which is different from the findings of previous results [5]. This may provide us with a clue that different stimulation methods are used at different acupoints and the mechanism of blood flow regulation in the local area may not be identical.

Some studies have suggested that the meridian system may contain a continuous channel [19] to facilitate signal transport in peripheral tissues [20, 21]. From this perspective, the vascular response along the meridian is intrinsically relevant. However, the results of this study showed that the blood flow response caused by different stimulation methods did not transmit along the meridian direction. These results are consistent with those of our previous study [22].

In this study, all stimulations were performed manually. Although the clinical practice was simulated, it could not guarantee the homogeneity of each stimulation, resulting in a certain degree of bias in the results. In addition, the sample size in this study was small, limiting the applicability of the results.

## 5. Conclusions

There was a difference in the response of blood perfusion at PC4 after acupuncture or moxibustion stimulation, which was related to the frequency V interval. However, this response was not observed at the surrounding points.

## Data Availability

The datasets presented in this study can be found in online repositories. The names of the repository/repositories and accession number(s) can be found at https://doi.org/10.6084/m9.figshare.16968352.v1.

## Supplementary Materials

Supplementary Materials Figure S: correlation analysis of random signals with a data length of 60000. (A) Random signal vector x. (B) Random signal vector y. (C) Histogram of signal x. (D) Histogram of signal y. (E) Pearson correlation coefficient changing with data frame, estimated using the toolbox provided by David J. Mack.
