# Supplementary material for: Pilot Study of Blood Perfusion Changes at PC4 and Its Surrounding Points Induced by Acupuncture and Moxibustion
Source: Evid Based Complement Alternat Med. 2021 Nov 26;2021:2431570. doi: 10.1155/2021/2431570 (PMC8641990; doi:10.1155/2021/2431570)
Supplement: Supplementary Materials — Figure S: correlation analysis of random signals with a data length of 60000. (A) Random signal vector x. (B) Random signal vector y. (C) Histogram of signal x. (D) Histogram of signal y. (E) Pearson correlation coefficient changing with data frame, estimated using the toolbox provided by David J. Mack. [file 2431570.f1.pdf]

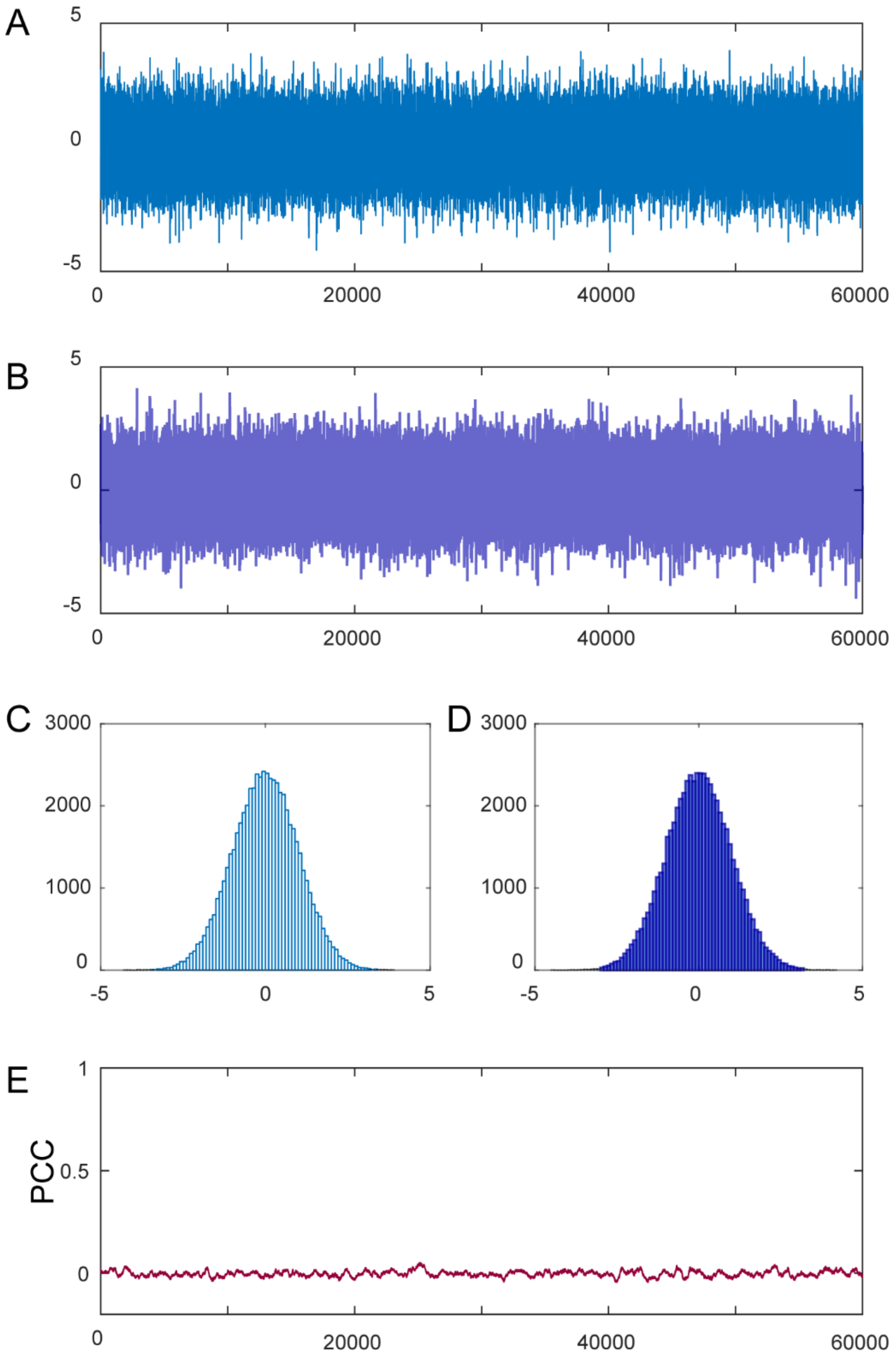

Figure S. Correlation analysis of random signals with a data length of 60000. (A) random signal vector x. (B) random signal vector y. (C) histogram of signal x. (D) histogram of signal y. (E) Pearson correlation coefficient changing with data frame, which is carried out with toolbox provided by David J. Mack[1]

1. D.J. Mack. "movcorr(x, y, k, varargin): Compute windowed correlation coefficient (https://www.mathworks.com/matlabcentral/fileexchange/65342-movcorr-x-y-k-varargin-compute-windowed-correlation-coefficient), MATLAB Central File Exchange. Retrieved May 24, 2021.."
